# High‐stakes, remote‐access, open‐book examinations

**DOI:** 10.1111/medu.14247

**Published:** 2020-06-25

**Authors:** Amir H. Sam, Michael D. Reid, Anjali Amin

## 1 WHAT PROBLEMS WERE ADDRESSED?

The coronavirus disease 2019 (COVID‐19) pandemic has led to unprecedented challenges in medical school assessments. Final‐year high‐stakes assessments have classically used closed‐book examinations (CBEs). Alternative methods of assessment such as open‐book examinations (OBEs) are emerging but are not routinely used in final‐year medical school examinations. The OBE encourages the use of problem‐solving skills more akin to those used in real life. There are currently limited data comparing OBEs with CBEs. A systematic review showed there was insufficient evidence to support the exclusive use of either CBEs or OBEs in assessment; however, the studies conducted to date have rarely looked at high‐stakes assessments as a result of concerns about the validity of OBEs.^1^

## 2 WHAT WAS TRIED?

In view of the restrictions put in place secondary to COVID‐19, we opted to use the two final‐year applied knowledge tests that had been scheduled to be used in CBEs as remote‐access OBEs. Candidates were able to access the examinations from anywhere in the world using any device with Internet access via an online platform. The papers were constructed from the United Kingdom Medical Schools Council bank of single best answer examination questions, which assess the candidate’s ability to integrate clinical reasoning and decision‐making skills. As the assessment aimed to assess the synthesis of knowledge rather than factual recall, there was no theoretical advantage to sitting the examination in an OBE rather than a CBE format. The psychometric analyses of the OBEs were compared with those of the written CBEs for the last 3 years. The OBEs were of the same duration as the previous CBEs. Only answers submitted to the online platform during the approved time frame of the OBEs were accepted. The order of the items in the OBEs was randomised for all candidates to mitigate against the risk for conferral.

## 3 WHAT LESSONS WERE LEARNED?

The median mark for the OBEs was identical to the median mark for the last 3 years of CBEs. The average discrimination of the OBEs was comparable with that of the CBEs when measured by mean point biserial. The number of distinctions and merits awarded were similar to those of previous years. Furthermore, the Cronbach’s alpha for the OBEs remained above 0.80, demonstrating good reliability that was similar to that of the CBEs over the last 3 years.

To the best of our knowledge, this is the first time that a final‐year, high‐stakes medical school examination has been administered both remotely and using an open‐book format. Our results suggest that concerns about the use of OBEs in high‐stakes assessments may be unfounded and that remote OBEs present a viable alternative to traditional CBEs if the questions appropriately assess the integration and synthesis of knowledge rather than factual recall. We propose that a combination of remote‐access online OBEs and proctored CBEs might be used in the future to strike a balance between the authenticity and validity of assessment programmes. Further studies should examine the value of online proctoring in high‐stakes OBEs.

Delivering the OBEs effectively required having the appropriate people, platform and processes in place. A dedicated team was available throughout the examinations to address any issues encountered by students. We developed processes for addressing common problems such as Internet connectivity issues. Having an appropriate online assessment platform was also crucial. Candidate feedback was positive and accepting of the changes to expected assessments in light of the unprecedented circumstances.
